# OA14 A case of scleromyxedema with muscle involvement including myotonia and dysphagia

**DOI:** 10.1093/rap/rkac066.014

**Published:** 2022-09-28

**Authors:** Marwa Mohareb, Adaeze Ugwoke, Nagui Gendi

**Affiliations:** Basildon and Thurrock Hospital, Basildon, United Kingdom; Basildon and Thurrock Hospital, Basildon, United Kingdom; Basildon and Thurrock Hospital, Basildon, United Kingdom

## Abstract

**Introduction/Background:**

Scleromyxedema is a rare, para-neoplastic, progressive condition of the Lichen myxedematous family. It is clinically characterized by generalized papules with skin infiltration and underlying systemic disorders, which may be fatal as it still constitutes a therapeutic dilemma.

**Description/Method:**

We are reporting a rare case of scleromyxedema presented to the rheumatology department mainly with dysphagia, weight loss, lower limb weakness and myotonia under dermatological and haematological review.

Case description: In January 2011, a 49-year-old man who was diagnosed year earlier with papular mucinosis and paraproteinemia-M band 8g/l (Monoclonal gammopathy of undetermined significance (MGUS)) presented with dysphagia mainly for solid, weight loss about 10 kg over the period of three months without other B symptoms and stiffness mainly in the hands and hips when walking or opening a jar described as slow relaxation following contraction.

On examination he looked well with full upper limb muscle strength. Scleromyxedmatous skin lesion in the form of multiple painful erythematous plaques and grouped papules involving his knees, elbows, dorsum of the hands and feet were appreciated. There were no telangiectasias or calcinosis. Neurological examination was unremarkable without muscle wasting or fasciculations, but there was a slow release of grip. Chest, heart, and abdominal examination was normal.

There was marked improvement of his skin lesion on 50mg thalidomide up to 150 mg daily, however his condition, including the skin lesions fluctuated in severity. Overall, he exhibited a benign course. The myotonia seemed to improve over time.

**Discussion/Results:**

Investigations showed raised creatine kinase at 1200 with normal full blood counts, normal renal, liver, calcium, CRP, ANA, ANCA and thyroid function test, normal Anti GAD, normal anti-voltage-gated potassium channel antibodies, normal barium swallow, unremarkable CT scan of chest, abdomen, and pelvis. Bone marrow biopsy ruled out plasma cell proliferation, M band always stable at 8 to 10 g/l. There were typical myotonic discharges on EMG with normal NCS. Normal MD and PROMM at ZNF9 genes.

Muscle biopsy showed several groups of very small atrophic fibres and patchy fibrosis with attempts of regeneration with PAS positive vacuoles deposition within the muscle fibres, replacing in places the majority of sarcoplasm with evidence of increased fibroblastic activity.

Over three to four years post diagnosis he developed only mild weakness in shoulder abduction and neck flexion MMT8 77/80, his CK remained high with a range of 900 to 3000 U/L. His weight stabilized.

Discussion: We present a rare case of scleromyxedema-associated with dysphagia, myotonia and muscle weakness. These symptoms in combination with cutaneous lesions raise the alternate diagnostic possibilities of systemic sclerosis-associated myositis, dermatomyositis, and myxedema. The diagnosis of scleromyxedema can be missed in this setting owing to the rarity of the disease. Histology can help make a definitive diagnosis; in our case mucinous material were demonstrated in the muscles as well as the skin lesions.  Although cases of scleromyxedema remain rare, a better understanding of its mechanisms of action could have implications for the research and treatment of autoimmune conditions. As our case several authors have reported rheumatic features in association with scleromyxedema in the form of erosive seronegative rheumatoid like arthropathy, sicca complex, dysphagia with oesophageal aperistalsis and sclerodactyly with acrolysis.

**Key learning points/Conclusion:**

The pathogenesis of systemic findings in scleromyxedema remains unknown although aggressive therapy mainly in the form of corticosteroid, thalidomide and IVIG may provide disease control, the prognosis remains guarded due to the systemic complications and high relapse rate. We need further studies to look at the link between scleromyxedema and systemic features.

